# Descriptive Epidemiology of Uruguayan Adults’ Leisure Time Physical Activity

**DOI:** 10.3390/ijerph15071387

**Published:** 2018-07-02

**Authors:** Javier Brazo-Sayavera, Gregore I. Mielke, Pedro R. Olivares, Luciano Jahnecka, Inacio Crochemore M. Silva

**Affiliations:** 1 Instituto Superior de Educación Física, Universidad de la República, Rivera 40000, Uruguay; 2 Polo de Desarrollo Universitario EFISAL, Universidad de la República, Rivera 40000, Uruguay; jahnecka2@gmail.com; 3 Centre for Research on Exercise, Physical Activity and Health, School of Human Movement and Nutrition Sciences, The University of Queensland, St Lucia, Brisbane, QLD 4072, Australia; g.ivenmielke@uq.edu.au; 4 Postgraduate Program in Epidemiology, Federal University of Pelotas, Pelotas 96020-220, Brazil; icmsilva@equidade.org; 5 Instituto de Actividad Física y Salud, Universidad Autónoma de Chile, Talca 1670, Chile; polivaress@uautonoma.cl; 6 Postgraduate Program in Physical Education, Federal University of Pelotas, Pelotas 96020-220, Brazil

**Keywords:** South America, surveillance, noncommunicable diseases, physical activity, adults

## Abstract

Policymakers rely on information for describing and monitoring levels of physical activity among the population. However, in Uruguay there is no research presenting physical activity practices nationwide. The present study aims to describe the leisure time physical activity levels and their unequal distribution among Uruguayan adults. Data from the 2014 Uruguayan National Health Survey (*n* = 3543 adults aged > 15 years) were analysed. Physical activity was measured by questionnaire, with participants reporting the number of days and time spent doing physical activity during leisure time in a typical week. Only 25.1% of the participants met the international recommendations for physical activity. Males were twice as active as females in early adulthood in terms of time. The absolute socioeconomic gap between the poorest and wealthiest income quintiles was around 20 percentage points, and participants with the highest physical activity levels were within the wealthiest and highest-educational levels. A low proportion of the population met the proxy of the international recommendations for physical activity. Important socioeconomic inequalities have been found in physical activity practices and must be considered in public health interventions.

## 1. Introduction

Physical activity (PA) is defined as bodily movements produced by skeletal muscles that require energy [1]. PA is a crucial component in the prevention and treatment of non-communicable diseases (NCDs) [2,3,4]. Annually, it is estimated that worldwide more than 5 million deaths could be attributed to physical inactivity [5]. Additionally, physical inactivity is responsible for a substantial economic burden, with a total worldwide cost ranging from INT$ 67.5 to INT$ 145.2 billion (combining direct and indirect costs) [6]. Despite this evidence, the prevalence of physical inactivity is still high among many populations. Estimates from 123 countries indicate that nearly a quarter (23.3%) of adults do not meet the current guidelines of 150 min of moderate-to-vigorous PA per week [7]. Therefore, given its health consequences and high prevalence, physical inactivity has come to be considered a significant public health problem globally [8].

Descriptive data providing current PA levels and temporal trends are essential for policymakers to implement effective non-communicable disease prevention programmes based on PA promotion [9]. In Latin America and Uruguay, 31.1% and 31.7% of adults are considered active (respectively) [10]. However, for most of these data, and particularly in Uruguay, available estimates are focused solely on the overall population. In this context, descriptive epidemiology studies are relevant in order to identify social inequalities and to better understand which groups have been left behind by public strategies for PA promotion.

To date, there is no evidence in the literature on PA level and its relation to socioeconomic inequalities in a national representative sample in Uruguay; this precludes the establishment of public health policies to tackle low PA levels [11]. However, in 2014 Uruguay set up a representative national health survey (Ministerio de Salud Pública, 2016), which included PA assessment among adults. Therefore, the present study aimed to describe overall PA levels and their relation to socio-demographic inequalities based on a nationally representative survey among Uruguayan adults.

## 2. Materials and Methods

Data from the Uruguayan National Health Survey, which was designed and conducted by the Ministry of Public Health of Uruguay in 2014 (from April to November), were analysed in this study.

A multiple stages sample selection was performed to include a representative sample of Uruguayans living in urban areas with a population of over 5000 residents. Initially, towns within Uruguay (excluding Montevideo, the capital) were selected probabilistically (proportional to their size) and stratified by Departments. Additionally, towns with a population between 5000 and 10,000 residents were sampled separately due to the lower likelihood of being selected. Census tracts and their associated households were systematically selected for each sampled town. All residents of the sampled households were eligible and invited to a face-to-face interview, which included a standardised questionnaire addressing socio-demographic information, health behaviours, and diseases. For the current analyses, only adults aged 15+ years were considered. All estimates were weighted according to the sampling process taking into account the probability of inclusion in the sample, adjustment for characteristics of those who did not answer the survey, and a calibration based on variables of the population of interest. All participants were informed prior to the interviewer’s visit. The information reported was considered official statistics by the Uruguayan National Institute of Statistics.

The questionnaire included a question about whether the participant did or did not undertake physical activity during leisure time. Additionally, leisure time PA (LTPA) was measured by questions assessing the number of days (three or more, one or two, and occasionally) and time spent on physical activities during leisure time (one hour or more, more than 20 min but less than one hour, and less than 20 min) in a typical week during the past 12 months. Participants were categorised into three groups according to their LTPA level: (1) low (less than 20 min, either occasionally or one to two days per week, or zero LTPA); (2) moderate (one or two days more than 20 min and three or more days less than one hour); and (3) high (at least one hour or more during three days or more). LTPA prevalence was considered as a new dichotomised variable with two categories: a proxy of the current international guidelines (high LTPA level) (World Health Organisation [7]) and the remaining options (low and moderate LTPA levels). This new variable was created to compare prevalence by region, socioeconomic status (SES), and educational level.

For the present analyses, the exposures/stratification variables used were sex (male/female), age (15–24; 25–34; 35–44; 45–54; 55–64; 65+ years), SES, and educational level. SES was categorised into five groups based on household asset index and analysed using principal component analysis (PCA), in which Q1 represents the poorest 20%, and Q5 represents the wealthiest 20%. Educational level was classified as: (A) uneducated and uncompleted primary education; (B) completed primary and uncompleted secondary education; (C) completed secondary and uncompleted higher education (tertiary level); (D) completed higher education (tertiary level). Based on the resultant sample distribution, educational levels A and B were merged to avoid small categories in the stratified analyses.

The prevalence of participants meeting PA recommendations by administrative areas (regions) was presented on a map. Crude prevalence ratio was calculated with 95% confidence intervals (95% CIs) and was presented for each of the independent variables. Double stratification was performed by (a) region and gender; (b) gender and age groups; (c) age groups and wealth quintiles, and (d) age groups and educational levels. The Equiplot graphical method (www.equidade.org) was used to visualise the differences in the prevalence of LTPA between age groups, education levels, and socioeconomic positions. All analyses were conducted in Stata 12.0.

## 3. Results

The analytical sample included 3543 participants from 74 different towns or cities in 13 administrative areas (regions). The response rate was 81.8% and our estimates can be extrapolated to the urban areas of Uruguay. Over half of the participants were female (57.2%); 18.6% and 18.4% were 15–24 years and 65+ years, respectively; and almost half of participants (48.1%) reported no education, uncompleted primary education, or having only completed primary education (Table 1).

Nearly half of participants were classified as being in the low LTPA category (males: 49.4%; females: 62.9%). Males reported a higher prevalence of moderate to high LTPA (19.3% and 31.3%, respectively) than women (16.7% and 20.4%, respectively). Prevalence of high LTPA levels decreased with age and the prevalence of low LTPA levels increased correspondingly; however, the proportion of moderate LTPA did not vary according to age group (Table 1). A large portion of participants reported they did not do any LTPA (50.8% in total; males: 45.1%; females: 55.0%). Analysing these responses by sex and age group, it was found that the prevalence of participants who did not do any LTPA ranged from 27.2 to 55.5% for men (15–24 and 55–64 age groups, respectively) and from 47.8 to 60.5% for women (45–54 and 25–34 age groups, respectively); it was higher in females in all age groups (Table S1).

Participants from the poorest quintile were more likely to report low LTPA (69.5%; 95% CI: 64.4–74.1) while the two wealthiest quintiles (Q4 and Q5) were more likely to report high LTPA (32.1%; 95% CI: 28.0–36.5 and 30.2%; 95% CI: 26.4–34.3, respectively). For the educational component, individuals with no/incomplete studies or only primary studies completed presented a higher prevalence of low LTPA (71.9%; 95% CI: 54.4–84.6 and 65.6%; 95% CI: 62.5–68.5, respectively) and those with a higher level of completed education reached a higher percentage of high LTPA (30.5%; 95% CI: 26.8–34.7).

**Figure 1:**
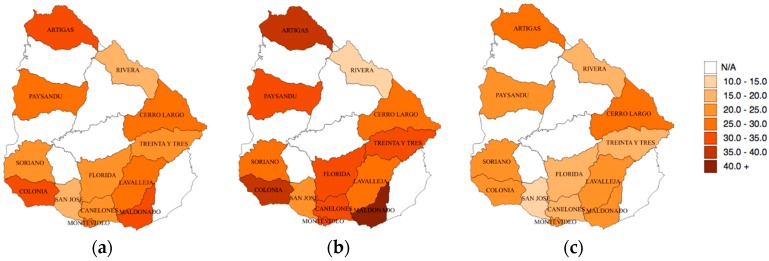
(**a**) General prevalence map of recommended physical activity (PA) in Uruguay; (**b**) Male prevalence map of recommended PA in Uruguay; (**c**) Female prevalence map of recommended PA in Uruguay. Colours represent prevalence in percentage. N/A: Not applicable.

The prevalence by region of people meeting the recommendations for weekly LTPA is shown in Figure 1. The region of Maldonado (Figure 1a) presented the highest LTPA prevalence (30.4%), while the region of Rivera presented the lowest prevalence (15.7%). The regional distribution for males (Figure 1b) was identical to the overall prevalence, but not for females, as the highest prevalence of women meeting PA recommendations was in Cerro Largo (28.2%) and the lowest was in San José (10.2%) (Figure 1c). The most significant difference identified between males and females was in Maldonado (females’ prevalence was 19.2 percentage points lower when compared to males’) and only in Rivera was the prevalence of recommended LTPA higher among females.

**Figure 2:**
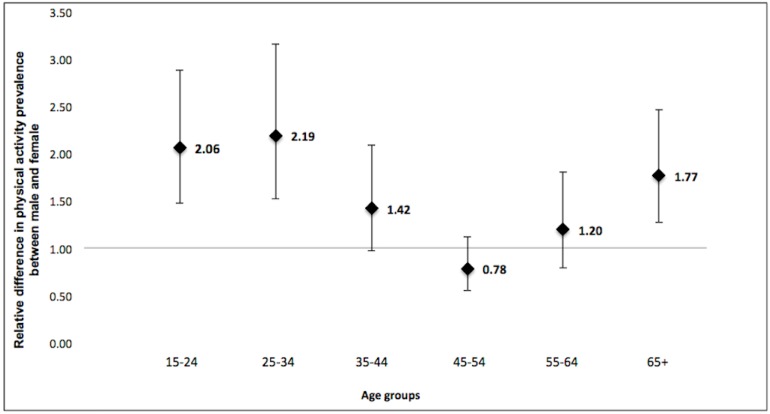
Relative difference in prevalence of recommended LTPA between males and females according to age groups.

Figure 2 shows the relative difference in prevalence of recommended LTPA between males and females in the whole country stratified by age groups. Relative difference in LTPA between males and females was higher among younger participants (15–34 years) and decreased up to the middle age (45–54 years) without identified differences. Among the elderly (65 years or more), the relative difference was observed again in the same direction (LTPA higher among males) but to a lower extent.

**Figure 3:**
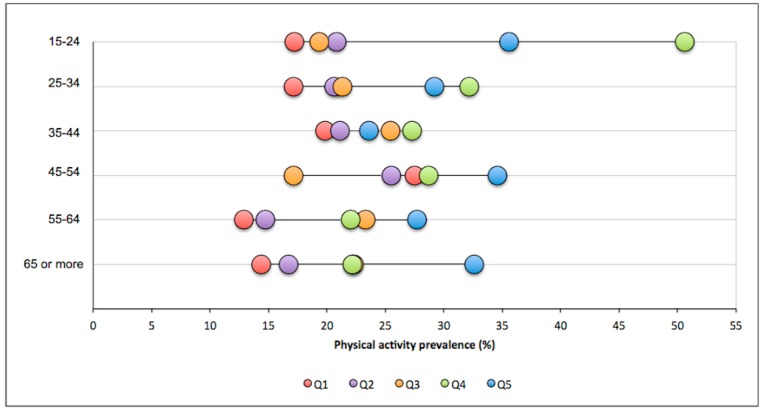
Differences in adherence to the PA recommendation guidelines in Uruguay according to socioeconomic status and age group. Q1: Quintile 1 (poorest); Q2: Quintile 2; Q3: Quintile 3; Q4: Quintile 4; Q5: Quintile 5 (wealthiest).

Double stratification of age groups and SES is presented in Figure 3. The poorest SES (Q1) presented the lowest prevalence of recommended LTPA in almost all age groups, while the wealthiest (Q5) presented the highest prevalence of recommended LTPA also in almost all age groups. The most significant gap between the poorest and wealthiest groups was in the age groups at the extremes of the age range (15–24 years and 65 years or more) and was less marked in the age group 35–44. The overall average gap between Q1 and Q5 was 12.5 percentage points. The poorest groups presented an increase in recommended LTPA prevalence until middle age (45–54) and a decrease after that. The wealthiest groups showed a decrease in prevalence for the first two age groups and an increase for the oldest age groups.

**Figure 4:**
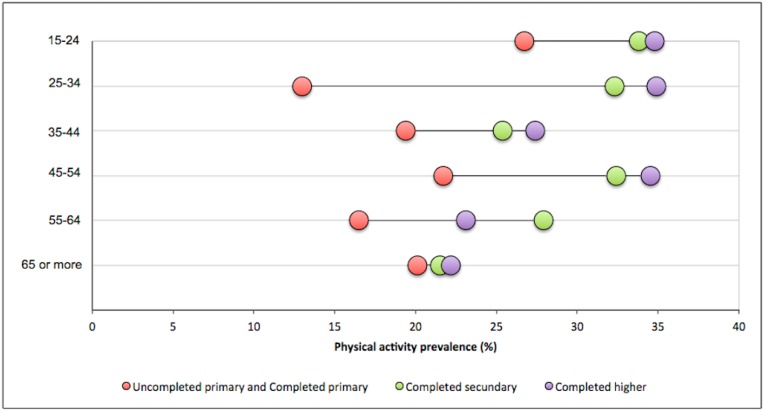
Differences in prevalence of adherence to the PA minimal weekly recommendation in Uruguay according to educational level and age group. Dots represent the different educational levels.

Educational levels A (uncompleted primary) and B (completed primary) were merged to show results by age groups in Figure 4. The lowest educational levels presented a lower recommended LTPA prevalence in all age groups, while the highest educational level showed a higher recommended LTPA prevalence in all age groups, except the one between 55 and 64. The widest gap among educational levels was found in the 25–34 age group and the smallest gap was in the oldest group.

## 4. Discussion

This study was the first to present LTPA data from the Uruguayan National Health Survey. It showed that more than half of Uruguayan adults reported low levels of LTPA. Overall, females were less active than males in almost every age group. Furthermore, LTPA level differed according to age group (with a lower proportion of participants reporting higher LTPA levels as age increases), socioeconomic status, and educational level (with higher LTPA levels reported among the wealthiest quintiles and higher educational levels).

Similar findings in terms of differences in LTPA by sex have been reported in studies from countries with different income levels [12,13]. Previous studies analysing data from 34 low-income and middle-income countries showed that time spent on LTPA is almost twice as long for men as for women [14]. Our analyses showed disparities by sex and age in Uruguay, with differences being more prominent in the youngest of the selected age groups, predominantly in the 15–24 and 25–34 age groups. These results are similar to those obtained by Azevedo et al. in a southern city in Brazil [15], although in their study the prevalence of participants who did not undertake any LTPA was marginally higher than ours for all age groups and in both sexes. 

Another study, sampling only one section of Uruguay’s population, reported differences between sexes, with the lowest scores being among women, retirees, the unemployed, and homemakers [16]. Therefore, we hypothesised that cultural and social factors, as well as the family structure or past inequalities in education opportunities, could affect women’s participation in LTPA [17]. In this regard, Brown [14] indicated that an increase in female participation in LTPA and sports is essential not only for health and quality of life, but also because it could positively impact economic development in different geographical, cultural, and political contexts. Following these indications, we recommend that Uruguayan policymakers develop new initiatives to promote LTPA in females, especially for women in the 15–24 and 25–34 age groups. The presented findings regarding low LTPA, viewed in combination with reported obesity levels in Uruguay, might help to explain why the highest prevalence of obesity in Latin America is among women under 20 [18].

In the present study, regional differences proved to be significant (although data from some regions were not available). Such differences could be due to different barriers identified among regions with higher or lower socioeconomic status [19]. Additionally, and comparably with other studies [15], differences according to socioeconomic status were found, with the wealthiest quintiles presenting the highest level of conformance with PA guidelines. On average, the wealthiest participants reported around 12.5 percentage points greater prevalence of recommended LTPA than the poorest. The lowest recommended LTPA prevalence being in the poorest group could be a result of lack of opportunities to access sports practices or places to perform PA, which represents a strong contextual factor influencing LTPA [20]. Hence, a lack of available public spaces to undertake PA could be a reason for this socioeconomic disparity because, as a result, people must search for structured activities (e.g., sport and fitness clubs), which require a higher financial investment [15]. 

It is also important to emphasise that the observed relationship between PA and socioeconomic status could completely change if we consider all domains of PA (including, in addition to leisure time, transport- and work-related activities). When all PA domains were included, previous studies reported the opposite association between socioeconomic status and PA levels [21]. In this regard, Celis-Morales [22] showed that Occupational PA accounts for 73% of total PA reported by the Chilean population in the Chilean National Health Survey, underlining the relevance of these other domains in measures of overall PA. This result also agrees with data from countries with high incomes such as Germany, in which PA prevalence among people with a lower educational level is higher overall but lower in their leisure time, which could be explained by their higher level of work-related PA [23]. 

Some limitations should be considered. Data used in this study are part of the Uruguayan National Health Survey and the sample process was limited to urban areas (rural areas represent around 5% of the population). In this survey, only questions relating to LTPA were included; this limitation did not allow for the calculation of PA in all domains. LTPA was assessed by considering a participant’s typical week, which may help to avoid recall bias but could also generate an idealised report, providing a scenario of LTPA practice in ideal conditions. Participants may also overestimate their LTPA due to the face-to-face method of interview [24]. Additionally, the included questions did not enable calculation of Metabolic equivalents from LTPA. Although a direct comparison against WHO PA guidelines was not possible, with the available information in this survey we calculated a proxy of the current WHO international guidelines in order to acquire essential information and enable further international comparisons. However, according to the structure of the questions and our operational definition some participants classified as practicing moderate levels of PA may reach WHO guidelines and may be classified as not meeting our proxy estimate. For future National Health Surveys, we recommend improving the PA module by adopting stepwise-approach guidelines comparable to other PA estimates worldwide. 

## 5. Conclusions

In conclusion, 50.8% of the adult population in Uruguay does not practice any LTPA, with levels being lower in women than in men. Only 30.1% of men and 20.1% of women reach the proxy of PA recommendations. Moreover, significant disparities were found in LTPA across regional, gender, socioeconomic, and educational categories. Interventions to address these inequalities should be implemented.

## Figures and Tables

**Table 1:** Sample description and leisure time physical activity (LTPA) prevalence according to socio-demographic variables.

Variables	N	%	Low	Moderate	High
Sex					
Male	1507	42.8 (40.7–44.9)	49.4 (46.1–52.6)	19.3 (16.9–22.1)	31.3 (28.4–34.4)
Female	2036	57.2 (55.1–59.4)	62.9 (60.1–65.6)	16.7 (14.8–18.9)	20.4 (18.3–22.7)
Age groups					
15–24	471	18.6 (16.7–20.5)	48.1 (42.5–53.6)	21.1 (16.9–26.1)	30.9 (26.2–36.1)
25–34	503	17.4 (15.8–19.2)	59.6 (54.2–64.8)	14.8 (11.5–18.9)	25.6 (21.3–30.5)
35–44	470	16.5 (14.9–18.3)	58.7 (53.1–64.2)	17.5 (13.6–22.1)	23.8 (19.5–28.7)
45–54	498	15.0 (13.6–16.6)	53.6 (48.1–58.9)	18.6 (14.7–23.2)	27.9 (23.3–33.0)
55–64	590	14.1 (12.8–15.5)	63.5 (58.4–68.3)	15.5 (12.3–19.3)	21.1 (17.1–25.7)
65+	1011	18.4 (17.0–19.8)	60.5 (56.5–64.3)	18.9 (16.1–22.2)	20.6 (17.4–24.2)
Socioeconomic status					
Q1 (poorest)	715	15.3 (13.9–16.9)	69.45 (64.4–74.1)	13.05 (10.0–16.8)	17.5 (13.8–22.0)
Q2	699	17.1 (15.6–18.8)	60.38 (55.3–65.2)	20.08 (16.3–24.5)	19.55 (15.8–24.0)
Q3	728	20.4 (18.7–22.2)	60.79 (56.2–65.2)	17.82 (14.7–21.5)	21.39 (17.9–25.4)
Q4	698	22.7 (20.9–24.6)	53.92 (49.3–58.5)	13.95 (11.1–17.4)	32.13 (28.0–36.5)
Q5 (wealthiest)	703	24.5 (22.6–26.4)	46.92 (42.5–51.4)	22.85 (19.4–26.8)	30.23 (26.4–34.3)
Educational level					
Uncompleted primary	69	1.6 (1.1–2.2)	71.9 (54.4–84.6)	17.1 (7.3–35.2)	11.0 (4.3–25.4)
Completed primary	1784	46.5 (44.4–48.7)	65.6 (62.5–68.5)	14.3 (12.3–16.6)	20.1 (17.6–22.9)
Completed secondary	981	29.3 (27.4–31.3)	53.6 (49.7–57.5)	17.0 (14.2–20.1)	29.4 (26.0–33.0)
Completed higher	709	22.6 (20.9–24.4)	43.2 (38.8–47.7)	26.2 (22.4–30.4)	30.6 (26.8–34.7)
Total	3543	N/A	57.1 (54.0–60.3)	17.8 (16.3–19.5)	25.1 (23.3–27.0)

Data expressed as % with 95% confidence interval (95% CI); Percentages calculated from weighted variables; N: absolute values (unweighted); Low: less than 20 min, either occasionally or 1–2 days per week (including zero LTPA); Moderate: one or two days with more than 20 min and three or more days with less than one hour; High: at least one hour or more during three days or more; Q: Quintile; N/A: Not applicable.

## Supplementary Materials

The following are available online at http://www.mdpi.com/1660-4601/15/7/1387/s1, Table S1: Prevalence of physical inactivity by gender and age groups; Table S2: Prevalence of recommended PA by regions; Table S3: Prevalence of conformance with proxy PA international recommendations by region, SES, and educational level.
