# Supplementary material for: Descriptive Epidemiology of Uruguayan Adults’ Leisure Time Physical Activity
Source: Int J Environ Res Public Health. 2018 Jul 2;15(7):1387. doi: 10.3390/ijerph15071387 (PMC6068702; doi:10.3390/ijerph15071387)
Supplement: Supplementary file 1 [file ijerph-15-01387-s001.pdf]

Supplementary Table 1. Prevalence of physical inactivity by gender and age groups.

Age groups	Male	Female	Total
15–24 years	27.2 (21.7–33.5)	52.4 (46.3–58.5)	40.8 (36.4–45.3)
25–34 years	43.0 (36.9–49.4)	60.5 (54.5–66.2)	52.3 (47.9–56.6)
35–44 years	45.7 (39.2–52.3)	51.8 (45.6–57.9)	48.9 (44.4–53.5)
45–54 years	49.8 (43.0–56.5)	47.8 (42.0–53.5)	48.6 (44.2–53.0)
55–64 years	55.5 (49.3–61.5)	53.9 (48.5–59.2)	54.6 (50.5–58.6)
65+ years	46.9 (41.8–52.0)	58.9 (55.0–62.6)	54.5 (51.4–57.6)
Total	45.1 (42.6–47.6)	55.0 (52.8–57.2)	50.8 (49.2–52.4)

Supplementary Table 2. Prevalence of recommended physical activity by regions.

Region	Total	Male	Female
Montevideo	25.9 (23.5–28.5)	31.7 (27.7–36.0)	21.8 (18.8–25.1)
Artigas	30.3 (21.7–40.7)	35.3 (21.1–52.7)	27.3 (17.1–40.6)
Canelones	23.7 (20.5–27.3)	30.1 (24.6–36.3)	19.5 (15.7–24.0)
Cerro Largo	28.2 (22.1–35.2)	28.2 (19.3–39.2)	28.2 (20.3–37.6)
Colonia	30.1 (24.0–36.9)	38.0 (28.7–48.4)	22.8 (15.6–32.0)
Florida	22.9 (17.9–28.8)	32.0 (23.4–41.9)	16.2 (10.8–23.5)
Lavalleja	26.6 (18.0–37.4)	28.6 (16.0–45.7)	25.0 (14.3–40.0)
Maldonado	30.4 (24.3–37.3)	40.9 (31.1–51.5)	21.7 (14.8–30.6)
Paysandú	27.8 (17.4–41.2)	33.3 (18.1–53.1)	22.2 (10.2–41.9)
Rivera	15.7 (11.8–20.8)	13.9 (8.5–21.8)	17.1 (11.8–24.1)
San José	16.0 (12.4–20.4)	24.1 (17.5–32.1)	10.2 (6.6–15.5)
Soriano	24.2 (17.4–32.5)	27.6 (17.6–40.5)	21.2 (12.9–32.8)
Treinta y Tres	24.3 (15.8–35.4)	31.4 (18.2–48.6)	17.9 (8.7–33.4)

Supplementary Table 3. Prevalence of conformance with proxy PA international recommendations by region, SES, and educational level.

	Age groups					
**Variables**	15–24 years	25–34 years	35–44 years	45–54 years	55–64 years	65+ years
**SES**						
Q1	17.2 (8.0–33.2)	17.1 (7.22–35.2)	19.8 (10.8–33.6)	27.5 (16.2–42.6)	12.9 (7.6–21.0)	14.4 (10.2–19.9)
Q2	20.8 (12.4–32.7)	20.6 (11.7–33.7)	21.1 (10.8–37.1)	25.5 (14.9–40.1)	14.7 (6.4–30.1)	16.7 (12.1–22.7)
Q3	19.3 (12.5–28.7)	21.3 (13.3–32.3)	25.4 (16.7–36.6)	17.1 (9.6–28.8)	23.3 (14.8–34.7)	22.3 (14.9–31.9)
Q4	50.6 (39.6–61.6)	32.2 (24.2–41.4)	27.3 (18.1–39.0)	28.7 (19.9–39.5)	22.0 (13.9–33.0)	22.2 (13.7–33.8)
Q5	35.6 (25.7–46.9)	29.2 (20.2–40.0)	23.6 (16.7–32.2)	34.6 (25.9–44.4)	27.7 (19.4 –37.8)	32.6 (23.1–43.7)
**Educational level**						
Incomplete primary to complete primary	26.7 (19.7–35.2)	13.0 (7.6–21.5)	19.4 (12.9–28.1)	21.7 (15.7–29.3)	16.5 (11.5–23.0)	20.1 (16.2–24.6)
Complete secondary	33.8 (26.4–42.2)	32.3 (24.1–41.7)	25.4 (18.5–33.9)	32.4 (23.7–42.7)	27.9 (20.0–37.4)	21.5 (15.0–29.8)
Complete tertiary	34.8 (24.7–46.4)	34.9 (27.3–43.4)	27.4 (19.6–36.8)	34.5 (25.2–45.2)	23.1 (15.2–33.6)	22.2 (14.1–33.0)

SES: socioeconomic status.
